# Characterization of PC-ABS and PETG Multi-Material Laminates Fabricated by MEX Method

**DOI:** 10.3390/polym18060763

**Published:** 2026-03-20

**Authors:** Mahalingam Nainaragaram Ramasamy, Ales Sliva, Akash Nag, Quoc-Phu Ma, Ondrej Hilser, Marie Heliova, Grazyna Simha Martynkova, Silvie Brozova, Jan Dizo

**Affiliations:** 1 Faculty of Mechanical Engineering, Vysoka Skola Banska-Technical University of Ostrava, 17. Listopadu 15/2172, 70800 Ostrava, Czech Republic; ales.sliva@vsb.cz (A.S.); ondrej.hilser@vsb.cz (O.H.); 2 Centre for Nanotechnology, Vysoka Skola Banska-Technical University of Ostrava, 17. Listopadu 15/2172, 70800 Ostrava, Czech Republic; 3 Faculty of Materials Science and Technology, Vysoka Skola Banska-Technical University of Ostrava, 17. Listopadu 15/2172, 70800 Ostrava, Czech Republic; 4 Faculty of Mechanical Engineering, University of Zilina, Univerzitna 8215/1, 010 26 Zilina, Slovakia; jan.dizo@fstroj.uniza.sk

**Keywords:** material extrusion, COMP (alternating PETG/PC–ABS laminate composite), PETG, PC–ABS, laminate, interfacial adhesion, mechanical properties

## Abstract

Material-extrusion (MEX) printing with automated filament switching enables single-build multi-material laminates, but interfaces between dissimilar polymers may govern failure. Here, monolithic PETG, monolithic PC–ABS, and an alternating PETG/PC–ABS laminate (COMP) with 0.2 mm laminae (4 mm total) were fabricated and benchmarked. Tensile behavior was measured using ISO 527-2 Type 1B specimens at 5 and 50 mm/min, complemented by three-point bending in horizontal/vertical orientations, unnotched Charpy impact (ISO 179), Shore D hardness (ISO 868), and SEM fractography. COMP delivered the highest horizontal flexural strength (159.82 ± 25.42 MPa), exceeding both single-material baselines, indicating improved bending load capacity in the preferred orientation. In Charpy impact, COMP absorbed more energy than PETG in the horizontal condition (0.86 ± 0.14 J vs. 0.57 ± 0.06 J) but remained below PC–ABS. In tension, COMP strength decreased by ~21–23% relative to PETG and by ~5–6% relative to PC–ABS at both speeds, consistent with interface-controlled damage. SEM revealed void-assisted crack initiation and interfacial debonding aligned with raster paths, highlighting interfacial strengthening and porosity reduction as key routes to improve tensile performance while retaining favorable flexural and impact response.

## 1. Introduction

Material extrusion (MEX) additive manufacturing (AM), commonly referred to as fused filament fabrication (FFF), has significantly evolved into a widely used method for producing polymeric parts with highly complex geometries in a short time. In recent years, it has gained attention for producing functional parts from engineering materials, beyond just prototyping [1]. Polyethylene terephthalate glycol (PETG) and polycarbonate–acrylonitrile butadiene styrene (PC-ABS) are two that stand out among the wide range of thermoplastics used for engineering applications. PETG offers flexibility, strong interlayer bonding, and chemical resistance, whereas PC-ABS provides durability with enhanced impact resistance and better heat tolerance than ABS [2,3].

The mechanical properties of PETG printed by MEX have been characterized in several studies, which exhibit strong sensitivity to process parameters such as raster orientation, layer thickness, and cooling conditions [4]. Tensile tests indicate that PETG typically demonstrates higher elongation at break than PC-ABS. Additionally, microstructural examinations highlight the importance of interlayer adhesion and the formation of voids, which influence the fracture behavior [5,6]. Regarding PC-ABS, existing reports highlight its superior thermal stability and toughness in comparison to ABS. However, they also acknowledge printing challenges associated with shrinkage, anisotropy, and residual stresses [7,8,9]. Characterizations of MEX-processed PC-ABS often reveal brittle fractures under sub-optimal conditions and strong dependence on printing parameters [10]. While these studies provide valuable insights into each material individually, there is almost no systematic work addressing their behavior in a layered combination. The combination of these two materials into laminated architecture offers an opportunity to obtain balanced mechanical properties with improved stiffness and better dimensional stability with minimal dimensional deviations.

Research into multi-material MEX has gained momentum in recent years, with most investigations focusing on polymer pairs such as PLA–ABS, PLA–PETG, or PETG with fiber-reinforced variants [11,12,13]. These works consistently show that interfacial bonding between dissimilar polymers has critical limitations in lamination, as differences in viscosity and glass-transition temperature restrict chain diffusion across boundaries and lead to weak interfaces [14,15]. Nevertheless, there remains a lack of standardized mechanical testing for fully printed PETG-PC-ABS blends as multi-material laminate composites. The lack of research is particularly significant given that both polymers are readily available as commercial filaments and that current multi-material printing systems can process them simultaneously. Recent desktop MEX platforms equipped with automated filament switching enable the production of multi-material laminates within a single build, shifting multi-material printing from a laboratory novelty to an accessible manufacturing option. However, designers still lack reliable, standardized mechanical property datasets and failure maps for common “off-the-shelf” engineering filament pairs [16,17,18].

This gap is especially critical for dissimilar polymers such as PETG and PC–ABS, where the mismatch in thermal transitions (PETG Tg ≈ 80 °C vs. PC–ABS Tg ≈ 115 °C) and melt/viscosity behavior can make the interfacial weld the dominant weak link. Although both PETG and PC-ABS are widely available as commercial filaments, little information is available on the behavior of their combination in alternating thin laminae under mechanical loading. Therefore, an investigation into the fabrication of PETG/PC-ABS multi-material as laminates that possess an equilibrium of strength, stiffness, and thermal resistance is justified.

Such laminates could be used in housings for electronics, protective guards, and interior automotive components where moderate mechanical loads combine with thermal or chemical demands. In addition to tensile response, many functional MEX components (e.g., housings, covers, brackets, and guards) operate under bending-dominated loading rather than pure uniaxial tension. Thus, including flexural performance metrics strengthens the design relevance of PETG/PC-ABS multilayer laminates within the broader landscape of multi-material MEX and polymer AM property benchmarking [16]. In addition, impact resistance is similarly critical for polymer components exposed to accidental drops, knocks, and intermittent shocks, where performance depends strongly on damage tolerance rather than only monotonic strength. For dissimilar-polymer laminates, rapid loading can activate failure pathways that may not be captured by tensile tests alone—such as interfacial debonding, crack deflection at layer boundaries, and energy dissipation through controlled delamination. In view of this, Charpy impact testing (ISO 179-1) can provide an interface-focused study in multi-material MEX and fracture/adhesion assessments. These tests help to establish a more complete and application-relevant performance baseline for PETG/PC-ABS layered systems [13,14,15]. Prior tribology studies on polymers report that scratch resistance can be related to fundamental mechanical parameters, including scratch hardness, and more recent work has proposed scratch hardness as a quasi-intrinsic descriptor of polymer scratch performance. Motivated by these findings, hardness testing is included in this study to provide a standardized, surface-focused indicator that complements the tensile, flexural, and impact results for PETG and PC-ABS laminates [19,20].

The objective of this study is to fabricate PETG/PC-ABS multilayer laminate coupons and evaluate their performance under tensile and quasi-static tests, an impact test, a hardness test, and a flexural test. The results are complemented by a scanning electron microscopy (SEM) analysis of fracture surfaces to investigate failure mechanics. The use of alternating 0.2 mm-thick laminate layers of both materials enables an investigation of the interfacial bond quality and potential failure pathways within a layered architecture. The study offers a foundational understanding of the trade-offs among strength, ductility, and interfacial adhesion in dissimilar-polymer multilayer laminates.

Following this Introduction, which provides the background of this study, Section 2 describes the materials that are used, the PETG/PC-ABS laminate fabrication process, the mechanical tests, and the SEM analysis. Section 3 reports the results and discusses the findings. Finally, the paper is concluded in Section 4.

## 2. Materials and Methods

### 2.1. Material

Two commercially available thermoplastic filaments were used: PETG and PC–ABS. The PETG filament was Fiberlogy EASY PETG, with a nominal diameter of 1.75 mm and white color, purchased from a commercial retailer (MaterialPro3D, Czech Republic). The PC–ABS filament was Fiberlogy PC–ABS, with a nominal diameter 1.75 mm and gray color, purchased from the same retailer. Lot or batch identifiers were not available on the packaging. To minimize moisture-related variability, both filaments were dried for 8 h at 60 °C using an AMS 2 Pro filament dryer manufactured by Bambu Labs, Shanghai, China and printing was performed immediately after drying. The filaments were stored in a sealed dry environment between prints. The basic mechanical properties of both PETG and PC-ABS provided by the manufacturers are shown in Table 1.

### 2.2. Printing Methodology

**Figure 1:**
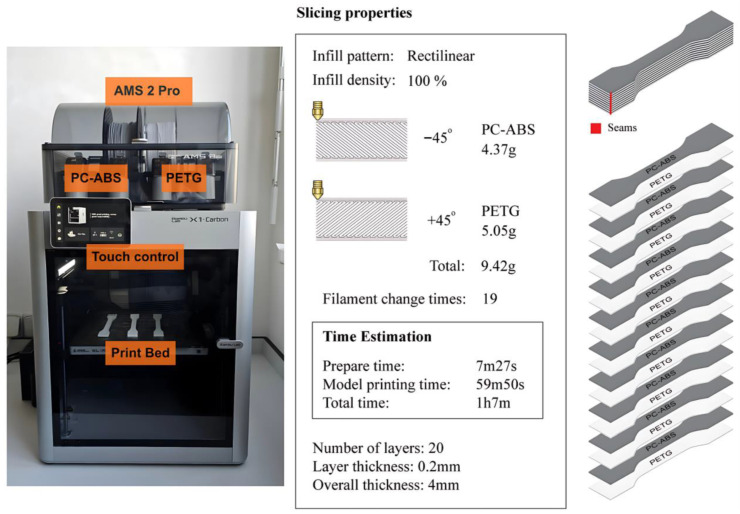
Bambu lab X1C 3D printer (**right**) and slicing properties of PETG and PC-ABS multi-material laminates (**left**).

All coupons were fabricated using a Bambu Lab X1 Carbon 3D printer manufactured by Bambu Labs, Shanghai, China with AMS 2 Pro and nozzle diameter of 0.4 mm (Figure 1), which is capable of processing multiple filaments in a single build. The printing parameters of PETG, PC-ABS, and their composite are shown in Table 2.

After several iterations to address the thermal and chemical incompatibility between PETG and PC-ABS, an alternating multilayer PETG/PC-ABS laminate was fabricated (hereafter, COMP). The laminate comprised 20 layers printed with a layer thickness of 0.2 mm (total thickness of 4 mm), starting with PETG on the build plate and alternating PETG and PC-ABS through the thickness. Coupons were printed at 100% infill using a rectilinear pattern to minimize porosity-related effects. Printing was performed in a closed chamber at 60 °C to maintain a controlled environment and reduce warping.

During COMP printing, PETG was extruded at 220–250 °C and PC-ABS at 260–270 °C; during filament changes, the nozzle temperature was held at 270 °C, and an increased purge was applied to reduce cross-contamination and clogging risk. The build plate temperature was set to 70 °C, and a glue layer was applied to improve adhesion and reduce warping.

### 2.3. Testing Methodology

#### 2.3.1. Tensile and Quasi-Static Test

**Figure 2:**
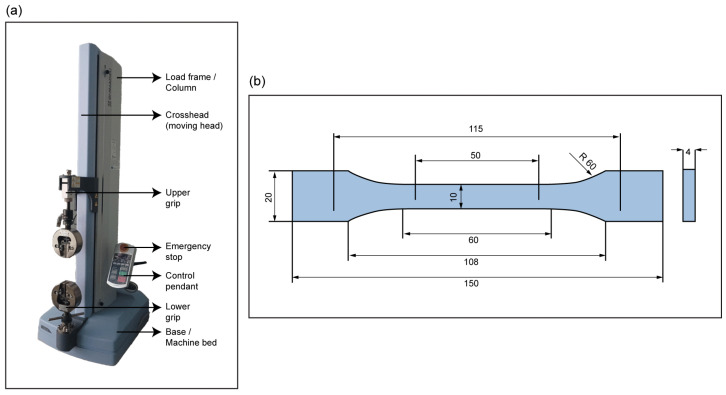
(**a**) Shimadzu EZ-LX UTM; (**b**) Dog-bone-shaped Type 1B standard coupons according to ISO 527-2 (unit: mm).

Mechanical testing was conducted in accordance with ISO 527-2 [22], using a Type 1B standard for both the tensile and quasi-static tests, as shown in Figure 2. Dog-bone-shaped coupons with standard dimensions were printed in all three materials: pure PETG, pure PC-ABS, and a multi-material laminate with 100% infill for comparison.

Tests were conducted on a Shimadzu EZ-LX UTM, with a stroke of 740 mm and a 5 kN load cell, under displacement control at a crosshead speed of 50 mm/min for tensile tests and 5 mm/min for quasi-static tests to evaluate possible delamination [23,24]. At least five replicates were tested for both tensile and quasi-static tests per configuration to ensure statistical reliability, as specified in ISO 527-2.

#### 2.3.2. Impact Test

**Figure 3:**
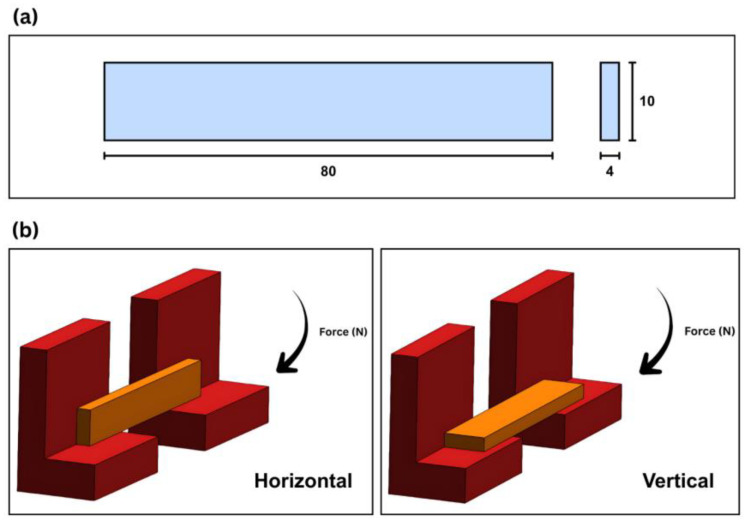
(**a**) Charpy impact specimen dimensions and (**b**) testing orientation, with the horizontal configuration shown on the (**left**) and the vertical on the (**right**).

Figure 3a presents the specimen dimensions used for the Charpy impact study, with a length of 80 mm, a width of 10 mm, and a thickness of 4 mm. Charpy impact testing was performed in accordance with ISO 179 without a notch to evaluate the impact energy absorption of the printed coupons [25]. In addition to reporting the absorbed impact energy, Wc (J), the unnotched Charpy impact strength, acU (kJ/m^2^), was calculated by normalizing Wc with the original specimen cross-sectional area (b × h), where b = 10 mm and h = 4 mm. For the present specimen dimensions, acU (kJ/m^2^) = 25 × Wc (J). The three coupons were manufactured for each material per orientation—PETG, PC-ABS, and COMP—to enable a direct material comparison. The orientation was determined by the angle between the coupon and the hammer. When the coupon, along with the printed lamina architecture, was tested perpendicular to the hammer orientation, it was considered horizontal. If the hammer was placed parallel to the coupon, along with its laminae architecture, it was considered vertical.

Figure 3b presents the testing orientation considered in the study. The horizontal configuration is shown on the left side of Figure 3b, and the vertical configuration is shown on the right side of Figure 3b. These two orientations were selected to capture the effect of build direction on crack initiation and fracture propagation under impact loading.

#### 2.3.3. Hardness Test

**Figure 4:**
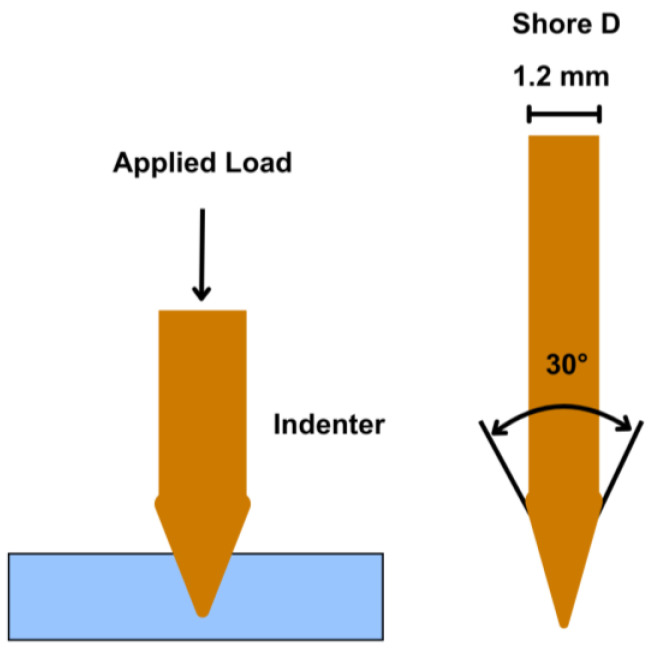
Shore D durometer hardness test, showing the loading setup on the (**left**) and the indenter geometry on the (**right**).

Figure 4 shows the Shore D durometer hardness test setup used in this work. Measurements were carried out according to ISO 868 using a Shore D durometer at a room temperature of 23.0 °C [26]. A vertical load was applied to drive the indenter into the specimen surface, as shown in Figure 4 (left). The indenter geometry is shown in Figure 4 (right), with a 30° tip angle, a diameter of 1.2 mm, and a tip radius of 0.1 mm.

#### 2.3.4. Three-Point Bending Test

**Figure 5:**
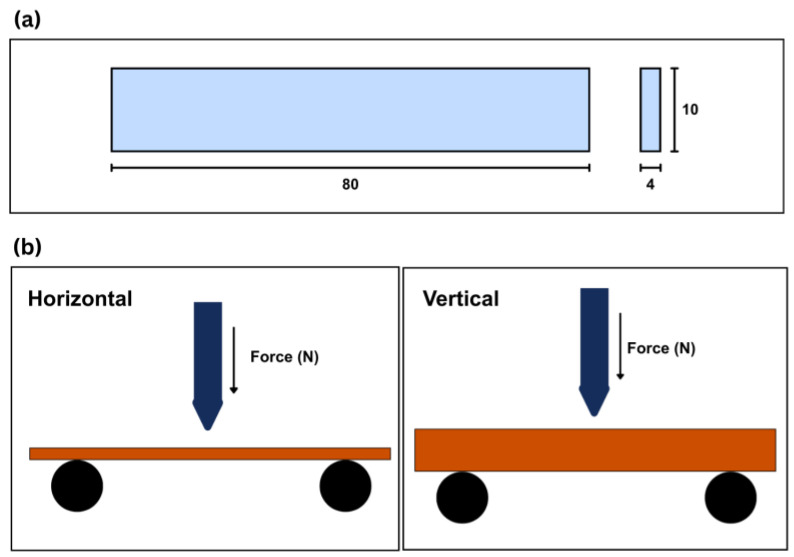
(**a**) Three-point bending test specimen dimensions and (**b**) testing orientation with horizontal on the (**left**) and vertical on the (**right**).

Three-point bending tests were conducted to evaluate the flexural response. Rectangular coupons had a length of 80 mm, a width of 10 mm, and a thickness of 4 mm. The support span was 40 mm. The loading-nose radius was 15 mm. The fixture used steel supports, as shown in the test setup, and the support contact geometry differs from standard roller definitions; therefore, the results are used for comparative evaluation between materials and orientations. Five coupons were tested for each material and orientation. The horizontal configuration tested coupons flat on the wider face, while the vertical configuration tested coupons on the edge. Coupons were fabricated in PETG, PC-ABS, and COMP to enable direct material comparison. Figure 5b illustrates the two testing orientations considered. In the horizontal configuration, coupons were tested flat on the wider face (perpendicular), while in the vertical configuration, coupons were placed parallel to the tool. These orientations were selected to capture the influence of build direction on flexural stiffness, peak load, and deformation behavior.

### 2.4. Fractography Observations

**Figure 6:**
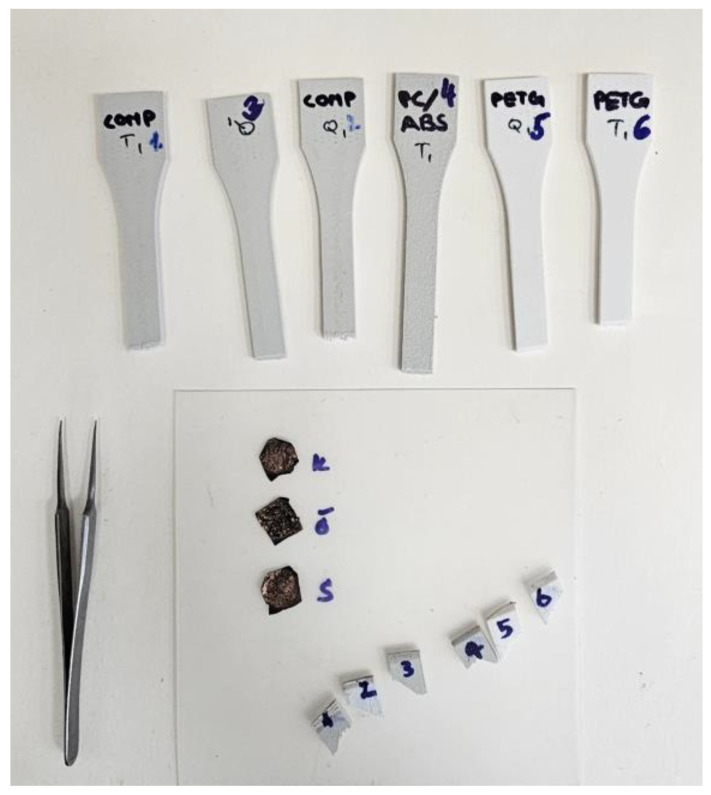
Coupons of PC-ABS, PETG, and their composite were prepared for SEM.

Once the tensile and quasi-static testing was completed, the fractured coupons were prepared for the microstructural analysis. The coupons closer to the fractured region were cut to 5 mm, and their surfaces were sputter-coated with 10 nm using a Quorum Q150 ES plus manufactured by Quorum Technologies, Laughton, East Sussex, UK as shown in Figure 6.

A JEOL JSM-7610F Plus SEM, manufactured JEOL Ltd., Akishima, Tokyo, Japan, was used to capture micrographs at 50× magnification. Additionally, for better assessment of the composite, 80× magnification was also employed. The fracture analysis primarily focused on the interfacial bond quality between the PETG and PC-ABS layers. These observations enabled correlation between the observed mechanical properties and fracture mechanisms, providing considerable insights into lamination failures.

## 3. Results and Discussion

### 3.1. Tensile and Quasi-Static Testing Results

The tensile and quasi-static tensile results are reported as mean values with standard deviation. For these tensile datasets, scatter was generally low (typically within 10% for most metrics), although elongation at break for PETG at 5 mm/min showed higher variability. A higher measure of elongation at break of PETG at 5 mm/min testing speed (approximately 16%) indicates more variability in its ductility, which is typical for polymers.

**Figure 7:**
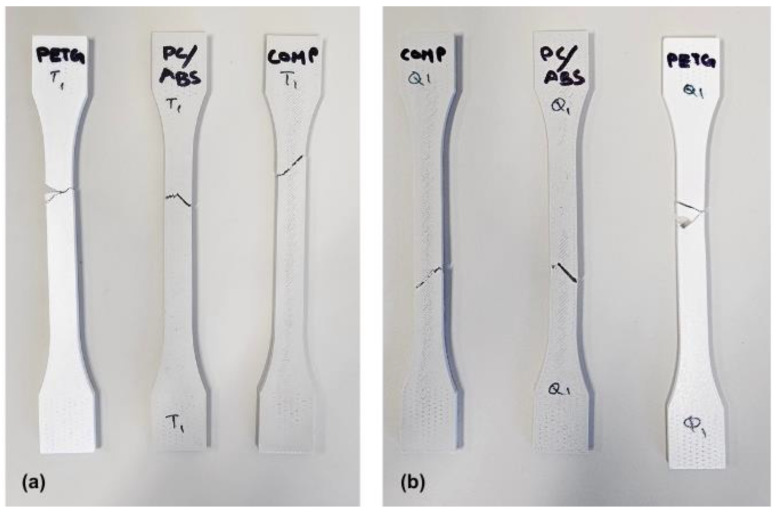
(**a**) Tensile test coupons of PC-ABS, PETG, and composite after fracture; (**b**) quasi-static test coupons of PC-ABS, PETG, and composite after fracture.

Compared with the reference base-material values (Table 1), the printed PETG and PC–ABS specimens show slightly higher ultimate strength. PETG exhibits higher ultimate strength and elongation at break than PC–ABS, consistent with prior reports [27,28]. Representative fractured specimens after tensile and quasi-static tests are shown in Figure 7. Across all materials, ultimate tensile strength increased with crosshead speed, consistent with strain-rate sensitivity in thermoplastics. Elongation at break showed material-dependent trends: PETG decreased slightly with speed, whereas PC–ABS and COMP increased modestly. Young’s modulus was broadly insensitive to speed within experimental scatter; any small differences may also reflect compliance effects because strain was inferred from crosshead displacement (no extensometer). Overall, these trends align with previous findings [27,28]. Table 3 and Table 4 shows the obtained values of Quasi-static and Tensile test results of PETG, PC-ABS, and their composite.

COMP exhibits lower ultimate strength, elongation at break, and Young’s modulus compared to the monolithic materials; however, successful fabrication demonstrates the feasibility of producing this multi-material laminate while maintaining its structural integrity. The overall performance follows PETG > PC–ABS > COMP across the measured tensile properties, and the difference between PC–ABS and COMP is relatively small. Since COMP comprises PETG and PC–ABS, this indicates that its tensile response is governed by the weaker constituent (PC–ABS) in the present laminate configuration.

### 3.2. Charpy Test Results

Charpy impact performance is governed by rapid energy dissipated by plastic deformation, crack deflection, and microstructural toughening mechanisms before complete fracture [25]. When normalized by cross-sectional area, the corresponding unnotched Charpy impact strength values are au = 14.25 ± 1.50 and 21.25 ± 1.50 kJ/m^2^ for PETG (horizontal and vertical), 43.50 ± 1.75 and 57.25 ± 4.50 kJ/m^2^ for PC-ABS, and 21.50 ± 3.50 and 21.25 ± 1.75 kJ/m^2^ for COMP. These values indicate a strong orientation effect for PC-ABS and PETG but not for COMP. Quantitatively, the vertical orientation improves W_c_ by approximately 32% for PC-ABS (2.29 J vs. 1.74 J) and by approximately 49% for PETG (0.85 J vs. 0.57 J).

**Figure 8:**
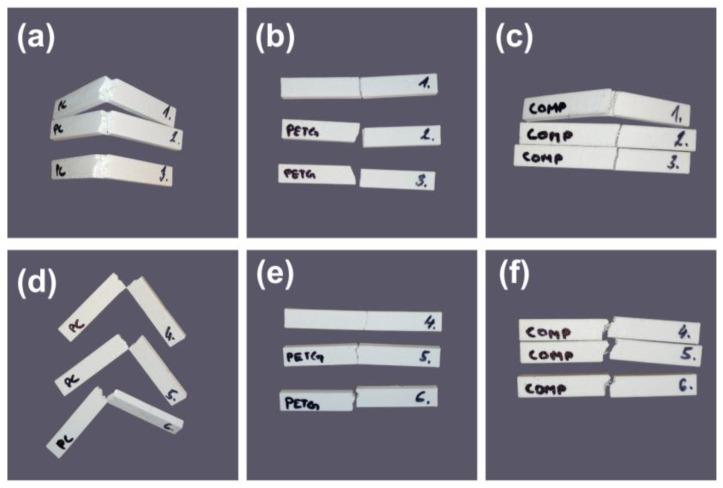
(**a**–**c**) Charpy test PC-ABS, PETG, and composite coupons tested in the horizontal direction. (**d**–**f**) PC-ABS, PETG, and composite coupons tested in the vertical direction.

The pronounced orientation dependence observed for PC-ABS and PETG aligns with MEX anisotropy, wherein the preferred crack path varies with raster/layer configuration and the number of welded interfaces encountered during fracture. When an impact-induced crack must traverse multiple roads and welded interfaces, increased energy dissipation occurs, whereas crack propagation along weaker interlayer regions results in a reduction in the absorbed energy [29,30]. This phenomenon is clearly visible in the COMP sample tested in the coupons shown in Figure 8.

**Figure 9:**
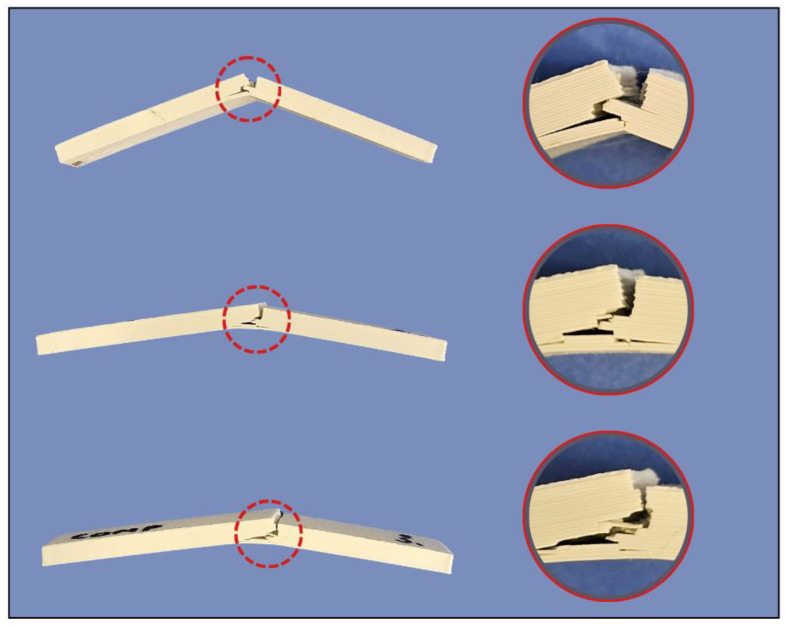
Delamination phenomena on composite coupons tested in the horizontal direction.

PC-ABS exhibits the highest impact energy because PC-ABS blends are designed for toughness, and the ABS rubber phase can activate multiple energy-dissipating mechanisms, such as shear yielding and crazing, which reduce interlayer sensitivity and increase impact resistance compared with more brittle thermoplastics [31,32]. A recent study on FDM-printed ABS–TPU blends reported improved ductility and fracture resistance, with morphology and thermal analysis highlighting the role of an elastomeric phase in governing fracture behavior and printed-layer integrity [33]. In contrast, the COMP results suggest a different limiting mechanism. Although COMP combines PC-ABS and PETG, it does not benefit from the vertical orientation and remains near 0.85–0.86 J, indicating that impact failure is likely governed by the weakest link in the architecture and arrangement of the laminae, most plausibly the dissimilar material interface and any local voids or imperfect healing at that interface. Interface-controlled fracture is widely reported in multi-MEX, where interfacial adhesion and thermal history can affect toughness irrespective of the constituent properties [29,30]. The repeatability in Table 5 is generally high, with most groups showing a coefficient of variation (CV) less than 10%, whereas COMP horizontal shows the largest scatter, consistent with the greater sensitivity of impact loading to local interfacial defects and void variability. In Charpy-tested COMP coupons in the horizontal orientation (samples 1–3), delamination initiation was observed (Figure 9) as localized partial separation along the PETG/PC–ABS interface, without complete layer peel-off. This behavior indicates that, once an impact-induced crack reaches the dissimilar interface, fracture propagation can be preferentially guided along the interfacial region, which is expected to be more sensitive to local voids, imperfect wetting, and incomplete interfacial healing than monolithic materials. Such interface-guided cracking is a recognized limitation in multi-material material-extrusion systems, where adhesion depends strongly on interfacial compatibility and diffusion-driven bonding mechanisms [34]. Furthermore, differences in material compatibility and thermo-mechanical mismatch (e.g., residual stresses linked to dissimilar thermal response) can further reduce effective interfacial resistance and promote interfacial crack deflection under dynamic loading. This interface-guided delamination provides a consistent explanation for (i) the limited orientation benefit for COMP (remaining near 0.85–0.86 J in both orientations) and (ii) the comparatively higher scatter in COMP (horizontal), since impact response becomes strongly controlled by local interfacial defect variability. These observations are based on post-test fracture-surface inspection; SEM characterization was not performed for Charpy coupons [35,36].

### 3.3. Hardness Test Results

Table 6 shows that PETG records the highest average hardness, 41.6 ± 3.4 Shore D, followed by COMP at 40.3 ± 4.9 Shore D, while PC-ABS exhibits the lowest average hardness at 37.4 ± 3.6 Shore D.

The difference between PETG and PC-ABS is about 4.2 Shore D points, indicating a meaningfully higher indentation resistance for PETG in this dataset. The scatter is modest for PETG and PC-ABS, with coefficients of variation of approximately 8.2% and 9.6%, respectively, whereas COMP shows the highest variability at approximately 12.2%, driven by a wider range of local readings from 32.5 to 45.5 Shore D.

This increased variability observed in COMP aligns with multi-material print applications, where local hardness may be affected by factors such as local thermal history, void population, and proximity to interfaces. Additionally, the indentation response can be influenced by the layered architecture rather than solely by a homogeneous polymer surface [37,38]. In a layered system such as COMP, the indenter not only probes the outermost skin but also the subsurface constraint, particularly when the overall thickness is limited; therefore, a stiffer supporting layer can reduce indentation depth and increase the apparent Shore D reading [26,39]. This provides a plausible explanation for why COMP exhibits a higher mean hardness than PC-ABS, despite PC-ABS being one of its constituents. The overall trend and magnitudes are also consistent with published FFF hardness studies, which show that printed polymer hardness depends on both the base polymer and the processing state, including moisture conditioning and print-induced microstructure [37]. In addition, recent blend studies report that Shore D hardness can remain relatively stable across certain polymer blend ratios even as other mechanical properties change, indicating that hardness does not always track tensile strength or impact toughness in a one-to-one manner [39].

### 3.4. Three-Point Bending Test Results

The three-point bending of material-extrusion polymers is primarily controlled by tensile-side crack initiation, the capacity of deposited layers and interlayer welds to transfer shear, and the presence of voids that intensify local bending stresses. Specimen orientation, therefore, influences both the maximum load and the work absorbed prior to failure, because the crack path can be forced to cut through roads and welds or, alternatively, can propagate along preferentially weakly bonded regions. This orientation-driven fracture steering is widely reported for fused-filament parts, where weld integrity is governed by temperature history, polymer interdiffusion, and entanglement development across bead boundaries [40,41,42,43]. The measured peak force, flexural energy, and flexural strength are summarized in Table 7.

#### 3.4.1. Coupons Tested in Horizontal Orientation

**Figure 10:**
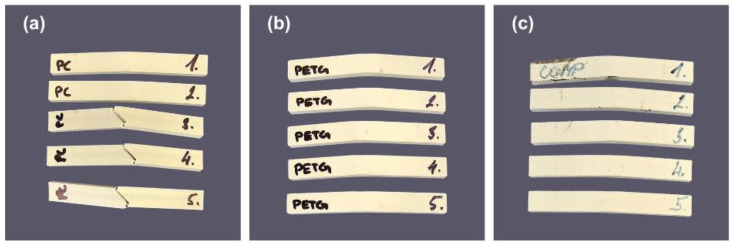
Representative post-test coupons after three-point bending in the horizontal orientation: (**a**) PC–ABS; (**b**) PETG; (**c**) COMP. PETG shows pronounced permanent deflection, while PC–ABS and COMP exhibit clearer crack initiation.

In the horizontal orientation (see Figure 10), COMP achieved the highest mean peak force of 426.2 N with a standard deviation of 67.8 N, closely followed by PC-ABS at 417.4 N with a standard deviation of 45.9 N, while PETG recorded 380.4 N with a standard deviation of 24.7 N. In terms of flexural strength, COMP again ranked highest at 159.82 MPa with a standard deviation of 25.42 MPa, marginally exceeding PC-ABS at 156.52 MPa with a standard deviation of 17.23 MPa, while PETG showed the lowest value at 142.65 MPa with a standard deviation of 9.26 MPa. This indicates that COMP provides an attractive bending load-bearing capability in the preferred horizontal orientation, where high strength is accompanied by strong resistance to tensile-side fracture initiation [42,43].

Energy absorption trends further support this interpretation. In the horizontal configuration, flexural energy ranked as COMP at 3078.1 N·mm with a standard deviation of 491.2 N·mm, PETG at 2899.6 N·mm with a standard deviation of 185.1 N·mm, and PC-ABS at 2622.2 N·mm with a standard deviation of 602.2. The comparatively high energy absorbed by COMP and PETG indicates greater work-to-failure during bending, consistent with more damage-tolerant behavior such as progressive microcracking, localized yielding, and crack deflection prior to unstable fracture [42,43]. Overall, in the bending test, scatter was higher for some conditions (notably COMP in the horizontal configuration and PC-ABS flexural energy), consistent with defect- and interface-sensitive failure.

#### 3.4.2. Coupons Tested in Vertical Orientation

A pronounced orientation sensitivity is evident for PC-ABS and COMP when moving from horizontal to vertical. PC-ABS shows a reduction in peak force of 26.8% and a reduction in flexural energy of 53.1%, while COMP shows a reduction in peak force of 16.6% and a reduction in flexural energy of 61.5%. The key observation is that, particularly for COMP, the loss in energy absorption is much larger than the loss in peak load. This pattern indicates that the vertical configuration promotes the earlier onset of unstable damage growth and reduced post-peak deformation capacity, even though the material can still sustain a relatively high maximum load. Such behavior is consistent with anisotropic failure in fused-filament structures, where the dominant mechanism can shift toward inter-road and interlayer separation when weld quality becomes the limiting factor under tensile-side loading.

**Figure 11:**
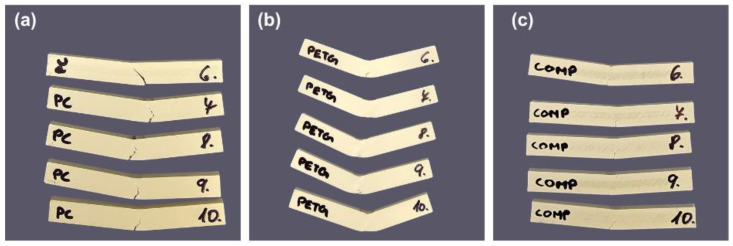
Representative post-test coupons after three-point bending in the vertical orientation: (**a**) PC–ABS; (**b**) PETG; (**c**) COMP. Vertical orientation promotes earlier cracking in PC–ABS and COMP, consistent with reduced post-peak energy absorption.

In contrast, PETG exhibits minimal orientation dependence in the present dataset. Peak force decreases only slightly, while flexural energy is essentially maintained and shows a small increase. This low sensitivity is consistent with the frequent observation of stable ductile bending in PETG-printed parts, in which strong interlayer bonding and higher ductility reduce the likelihood of brittle, weld-guided cracking and suppress orientation-driven swings in both strength and energy absorption [3,4]. These trends are also consistent with the post-test appearance in Figure 11, where PETG coupons exhibit pronounced permanent deflection without complete separation, while PC-ABS and COMP show clearer crack formation and earlier fracture features, particularly in the vertical set [42,43].

The response of COMP can be rationalized by its laminate-like architecture. Multi-material extrusion is frequently limited by interface-controlled failure when chemical compatibility and thermal history restrict chain diffusion and entanglement across dissimilar polymer boundaries [44]. Under the vertical configuration, the stress state can increase the demand on interfacial shear transfer and promote premature interfacial debonding. This provides a consistent explanation for why COMP remains competitive in peak force and flexural strength in the vertical orientation, yet exhibits a marked reduction in flexural energy, meaning that the specimen reaches peak load but dissipates substantially less work before unstable fracture. This interpretation aligns with established fusion-bonding physics in material extrusion and with published observations that weak interfacial regions become preferential crack paths under tensile-side loading, particularly in multi-material systems [44]. No macroscopic delamination was observed after bending and hardness testing based on visual inspection. Microstructural characterization using SEM was not conducted for these test conditions.

### 3.5. Fractography Analysis

**Figure 12:**
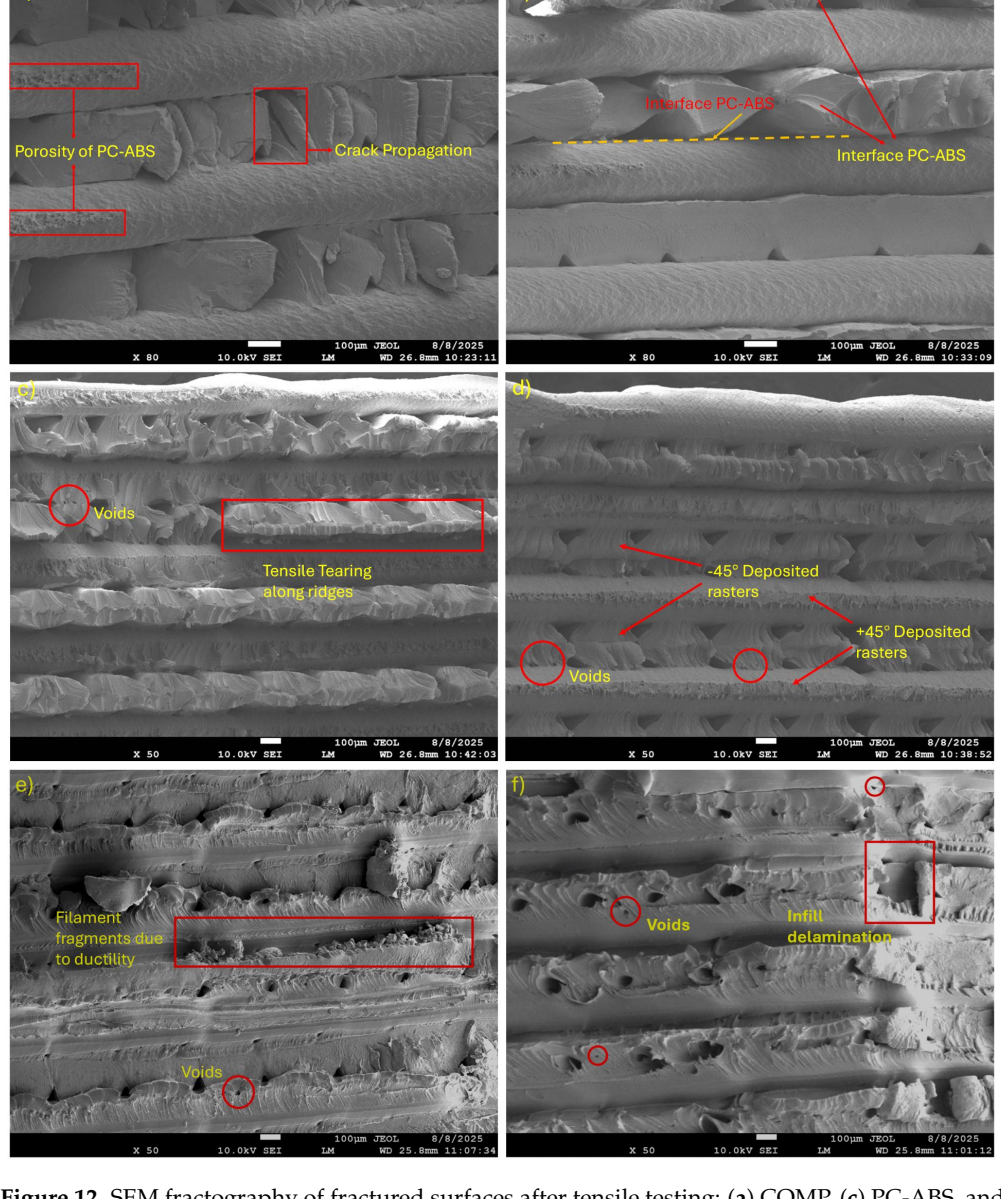
SEM fractography of fractured surfaces after tensile testing: (**a**) COMP, (**c**) PC-ABS, and (**e**) PETG; and after quasi-static tensile testing (5 mm/min): (**b**) COMP, (**d**) PC-ABS, and (**f**) PETG. SEM at 50×; composite imaged additionally at 80× for clearer interface observation.

No macroscopic delamination (complete layer peel-off) was observed in tensile and quasi-static tensile tests. SEM analysis was performed on these coupons to characterize fracture features and interface-related damage modes. Figure 12 provides the fractured surface analysis of all the tested samples and their combination, captured using SEM to analyze the failure mechanism using two distinct loading conditions, such as tensile and quasi-static tensile loading. Figure 12a shows a micrograph of the fractured composite sample, revealing the distinct layered morphology of the FDM-printed composite sample with the presence of porosity and crack propagation along the interlayer boundaries, suggesting interfacial weakness between the two deposited layers. The crack appears to follow the weakest zones, indicative of a predominantly brittle failure due to insufficient interlayer bonding or diffusion. Moreover, the poorly fused regions also serve as pre-existing defects or stress concentrators, thereby promoting preferred crack propagation paths. In the lower crosshead-speed (quasi-static) condition (Figure 12b), interfacial separation appears more progressive, with visible PC-ABS/PETG separation rather than the more abrupt fracture observed at the higher crosshead speed. The comparatively smoother surface is consistent with slower crack-growth kinetics and time-dependent viscoelastic relaxation. In both cases, fracture occurs through the laminate thickness without gross layer peel-off; however, the SEM features indicate that the PC-ABS/PETG interface and nearby pores act as preferential crack paths [45]. This suggests that interfacial adhesion is sufficient to avoid macroscopic delamination, but remains weaker than the bulk and therefore contributes to failure localization under tensile loading—consistent with dissimilar-polymer MEX systems where limited interdiffusion and thermal history constrain interfacial strength.

Figure 12c shows repeated tensile tearing along the layer ridges and voids along the inter-filament gaps. The concentration of the ridge edges indicates the crack growth following the deposited geometry, which is a typical failure mode in FDM when inadequate interlayer diffusion is present. Moreover, the presence of film-like necked regions between the rasters suggests limited local plasticity before failure. Further SEM observations of PC-ABS under tensile and quasi-static tensile loading reveal the cavitation of rubber-rich domains in the ABS phase, with microvoid coalescence producing dimples and drawn fibrils before final separation, consistent with rubber toughening mechanisms reported for PC-ABS blends and correlating with the previous study [46]. The micrograph of the fractured surface of the PC-ABS sample (Figure 12d) under quasi-static tensile loading clearly shows the printing raster orientation (±45°). Also, the failure surfaces appear to align with those rasters. These fractures suggest an anisotropic mechanical response, as the cracks propagate preferentially along the rasters or between them, following particular orientations. Fracture surfaces further reflect sensitivity to loading rate, where a quasi-static test shows more ductile-looking ridges during fracture. In contrast, tensile test results promote flatter regions with even fracture along the same plane [47].

Under quasi-static tensile conditions, ruptures are more elongated and exhibit greater plastic tearing along the ±45° rasters. In the case of PETG samples (Figure 12e), increased necking or ductile characteristics, as well as filament fragments, are observable, along with some voids and incomplete infill adhesion. The fragmentation suggests that PETG exhibits greater inherent ductility than PC-ABS or the composite; however, failure still initiates at internal voids and regions with inadequate inter-raster contact. For quasi-static testing samples (Figure 12f), ruptured surfaces are smoother and, in some instances, exhibit more interfacial separation bands. Due to slower loading, the raster accommodates larger deformation within the plastic zone, but the presence of voids still initiates and causes failure. Overall, the weakest points of all the samples observed through SEM images were identified as interfacial layers and voids. Even in more ductile PETG samples, voids and insufficient filament contact initiated the fractures. Further, the ±45° raster pattern modulated and induced directional crack propagation. Comparing the two testing methodologies, quasi-static tensile (slower-loading) tests produced more progressive crack growth, with inter-raster separation near voids and larger plastic zones, than the standard tensile test, which caused localized tearing. This phenomenon has also been observed in several previous studies examining the dependence of the loading rate on the viscoelastic/ductile polymer response [48,49,50,51,52]. In general, the fracture surfaces indicated that the PETG samples exhibited the greatest extent of stretching and plastic zones, followed by the PC-ABS samples, which showed moderate tearing aligned with the ridges. Conversely, the composite samples demonstrated the least ductility, with crack propagation primarily characterized by interfacial debonding and porosity. This also supports and complements the quantitative elongation values obtained during tensile and quasi-static tensile testing, providing a more comprehensive understanding of the material’s deformation behavior under varying testing conditions.

## 4. Conclusions

This work benchmarks an alternating PETG/PC–ABS laminate (COMP) produced by material-extrusion (MEX) printing against monolithic PETG and monolithic PC–ABS, with emphasis on how laminate interfaces govern rate-dependent tensile response, bending-dominated performance, and impact damage tolerance.

- Under quasi-static tensile loading (5 mm/min), PETG delivered the highest ultimate strength and elongation at break (53.7 ± 0.8 MPa; 13.4 ± 2.1%), followed by PC–ABS (44.7 ± 0.8 MPa; 10.4 ± 0.3%). COMP exhibited the lowest strength and ductility (42.4 ± 1.1 MPa; 8.5 ± 0.6%), indicating a tensile-property penalty for the laminate architecture under slow loading. Young’s modulus showed a similar ordering (PETG: 932 ± 2 MPa; PC–ABS: 881 ± 9 MPa; COMP: 873 ± 9 MPa), confirming that the laminate did not provide additive stiffening and that tensile performance is limited by interface/defect sensitivity.
- At the higher crosshead speed (50 mm/min), ultimate strength increased for all materials, consistent with strain-rate sensitivity. PETG remained strongest (59.1 ± 0.4 MPa), with PC–ABS being intermediate (48.3 ± 1.1 MPa) and COMP being lowest (45.6 ± 1.2 MPa). The laminate retained a similar deficit at both rates (≈21–23% below PETG and ≈5–6% below PC–ABS), indicating that interfacial integrity and local defect population—not constituent strength—dominate the tensile response. Young’s modulus remained comparable within scatter (PETG: 897 ± 14 MPa; PC–ABS: 874 ± 10 MPa; COMP: 860 ± 13 MPa).
- Charpy impact testing showed PC–ABS to be the toughest (1.74 ± 0.07 J horizontal; 2.29 ± 0.18 J vertical). COMP absorbed more energy than PETG in the horizontal condition (0.86 ± 0.14 J vs. 0.57 ± 0.06 J), while remaining well below PC–ABS. Unlike PETG and PC–ABS, COMP showed negligible orientation dependence (≈0.85–0.86 J), consistent with an interface-limited impact response in which the dissimilar-material boundary governs crack path and energy dissipation. Post-test inspection further revealed delamination initiation in horizontally tested COMP coupons (localized interfacial separation without complete peel-off), supporting an interface-sensitive fracture pathway under dynamic loading.
- Shore D hardness followed PETG > COMP > PC–ABS (41.6 ± 3.4, 40.3 ± 4.9, and 37.4 ± 3.6, respectively), with COMP showing the highest scatter. This dispersion is consistent with layered architectures, where indentation response is influenced by subsurface constraint and proximity to interfaces/voids.
- In three-point bending (horizontal orientation), COMP achieved the highest peak force and flexural strength (426.2 ± 67.8 N; 159.82 ± 25.42 MPa), marginally exceeding PC–ABS (156.52 ± 17.23 MPa) and outperforming PETG (142.65 ± 9.26 MPa). However, strong orientation sensitivity emerged for PC–ABS and COMP: moving from horizontal to vertical produced large reductions in flexural energy (PC–ABS: −53.1%; COMP: −61.5%), indicating earlier instability and reduced post-peak deformation capacity when load transfer relies more strongly on inter-road/interlayer bonding.
- SEM fractography corroborated these trends. COMP fracture surfaces showed void-assisted crack initiation and preferential propagation along interlayer boundaries and the PETG/PC–ABS interface; at lower crosshead speed, interfacial separation appeared more progressive, whereas higher-speed loading produced a more abrupt fracture morphology. Overall, PETG exhibited the greatest plastic deformation, PC–ABS showed moderate raster-aligned tearing, and COMP displayed the least ductility, with failure dominated by interfacial debonding and porosity—consistent with interface-governed performance in the laminate architecture.

The MEX method presented here produces minimal material waste and applies energy only where required, lowering the carbon footprint as compared to conventional manufacturing methods, such as melt-blending or molding. The MEX method can also adapt to batch customization without the need for new blends or molds, thereby reducing overall setup costs. The limitations of this study and possible future work are discussed below.

**Limitations**

- Coupon-scale scope: The study is limited to standardized coupon specimens; the behavior of larger or geometrically complex parts (where thermal gradients and residual stresses may differ) was not investigated.
- Batch demonstrator (not mass production): The COMP laminate was tested under controlled batch fabrication; industrial-scale repeatability and throughput in terms of production rate were not assessed in this study.
- Single validated printing window: All specimens were fabricated using one validated parameter set (Table 2) to ensure a fair comparison, but a broader process window (e.g., temperature/speed/cooling sensitivity) was not systematically explored.
- Limited impact-test repetitions: Charpy impact testing was conducted with n = 3 per configuration, which is adequate for comparative screening but still limits statistical robustness for impact performance.
- Fractography coverage: SEM fractography was focused on tensile and quasi-static tensile fractures; detailed microstructural/fracture observations for bending and impact failures were not included.

**Future work**

- Scale-up feasibility studies to evaluate repeatability on larger parts and to identify practical manufacturing routes for higher-throughput production.
- Process-window and interface optimization, including controlled studies of nozzle temperature, cooling, print speed, and interlayer time to improve interfacial bonding consistency.
- Increase statistical robustness, particularly for impact behavior (higher n of at least five, statistical testing, and scatter analysis).
- Expand microstructural diagnostics by adding µCT-based void quantification and interface inspection for bending and impact fracture surfaces.
- Durability and service performance, including humidity/thermal conditioning, aging, and cyclic/fatigue loading to evaluate the long-term stability of the laminate interface.

## Figures and Tables

**Table 1:** Mechanical properties of PETG and PC-ABS from the manufacturer [3,21].

Properties	PETG	PC-ABS
Glass Transition	80 °C	115 °C
Density	1.27 g/cm^3^	1.07 g/cm^3^
Tensile Strength	51 MPa	42 MPa

**Table 2:** Bambu lab X1 Carbon 3D printer settings.

Parameters	PETG	PC-ABS	Composite
Nozzle Temperature	220–250 °C	260–270 °C	PETG layers: 220–250 °C; PC–ABS layers: 260–270 °C; nozzle held at 270 °C during filament changes
Bed Temperature	90 °C	100 °C	70 °C
Print Speed	50 mm/s	50 mm/s	40 mm/s
Cooling Fan	off	25% on	25% on (PC-ABS)/off (PETG)
Purge	~1.2 g	~1.2 g	~5.5 g
Infill	100%	100%	100%
Mechanical interlocking	Not used	Not used	Not used
Infill pattern	Rectilinear	Rectilinear	Rectilinear
Wall loops	2	2	2
Top and bottom shell layers	3	3	3

**Table 3:** Quasi-static test results of PETG, PC-ABS, and their composite.

5 mm/min	Ultimate Strength (MPa)	Elongation at Break (%)	Young’s Modulus (MPa)	Mass (g)
PETG	53.7 ± 0.8	13.4 ± 2.1	932 ± 2	~10.314
PC-ABS	44.7 ± 0.8	10.4 ± 0.3	881 ± 9	~8.461
COMP	42.4 ± 1.1	8.5 ± 0.6	873 ± 9	~9.595

**Table 4:** Tensile test results of PETG, PC-ABS, and their composite.

50 mm/min	Ultimate Strength (MPa)	Elongation at Break (%)	Young’s Modulus (MPa)	Mass (g)
PETG	59.1 ± 0.4	12.7 ± 0.9	897 ± 14	~10.314
PC-ABS	48.3 ± 1.1	11.8 ± 0.5	874 ± 10	~8.461
COMP	45.6 ± 1.2	9.4 ± 0.7	860 ± 13	~9.595

**Table 5:** Charpy test results of PETG, PC-ABS, and their composite.

Material	Horizontal W_c_ (J) (n = 3)	Horizontal acU (kJ/m^2^)	Vertical W_c_ (J) (n = 3)	Vertical acU (kJ/m^2^)	Mass (g)
PETG	0.57 ± 0.06	14.25 ± 1.50	0.85 ± 0.06	21.25 ± 1.50	~4.014
PC-ABS	1.74 ± 0.07	43.50 ± 1.75	2.29 ± 0.18	57.25 ± 4.50	~3.481 g
COMP	0.86 ± 0.14	21.50 ± 3.50	0.85 ± 0.07	21.25 ± 1.75	~3.759 g

**Table 6:** Hardness test results of PETG, PC-ABS, and their composite.

Material	Hardness Value
PETG	41.6 ± 3.4
PC-ABS	37.4 ± 3.6
COMP	40.3 ± 4.9

**Table 7:** Three-point bending test results of PETG, PC-ABS, and their composite.

Material	Orientation	Peak Force (N)	Flexural Energy (N·mm)	Flexural Strength (MPa)
COMP	Horizontal	426.2 ± 67.8	3078.1 ± 491.2	159.82 ± 25.42
COMP	Vertical	355.6 ± 51.5	1185.5 ± 195.6	53.34 ± 7.72
PC-ABS	Horizontal	417.4 ± 45.9	2622.2 ± 602.2	156.52 ± 17.23
PC-ABS	Vertical	305.6 ± 32.1	1230.2 ± 165.3	45.84 ± 4.81
PETG	Horizontal	380.4 ± 24.7	2899.6 ± 185.1	142.65 ± 9.26
PETG	Vertical	373.6 ± 2.7	2979.4 ± 166.9	56.04 ± 0.41

## Data Availability

The data supporting the findings of this study are available at Zenodo (doi:10.5281/zenodo.18472960).

## Abbreviations

The following abbreviations are used in this manuscript:

ABS	Acrylonitrile–butadiene–styrene
AM	Additive manufacturing
AMS	Automatic Material System
COMP	Alternating PETG/PC–ABS laminate composite
FFF	Fused filament fabrication
MEX	Material extrusion
MEX-AM	Material extrusion additive manufacturing
PC–ABS	Polycarbonate–acrylonitrile–butadiene–styrene
PETG	Polyethylene terephthalate glycol
SEM	Scanning electron microscopy
UTM	Universal testing machine
